# Falls Prevention Behaviors by Family Caregiver-Person Living with Dementia Dyads and Falls

**DOI:** 10.1093/geroni/igaf122.4260

**Published:** 2025-12-31

**Authors:** Victoria Panzer, Veronica Smith, Joanne Maher, Bayard Kohlhepp, Richard Fortinsky

**Affiliations:** Brookside Research & Development, Coupeville, Washington, United States; Brookside Research & Development, Coupeville, Washington, United States; Brookside Research & Development, Coupeville, Washington, United States; Brookside Research & Development, Coupeville, Washington, United States; University of Connecticut, Farmington, Connecticut, United States

## Abstract

We previously reported the successful use of the FallScape-D computer-tailored multimedia falls-prevention intervention (FallScape-D) for family caregivers of persons living with dementia (PLWD). We enumerated caregiver-reported falls prevention behaviors (FPBs) during the 4-6 week intervention. This study enrolled a small intentionally-varied PLWD-caregiver dyad cohort, delivered Fallscape-D, and followed dyads for up to one year.

**Methods:**

Eleven family caregivers (6 female; mean(SD) age=65.6(12.8)) delivered FallScape-D, consisting of daily caregiver presentation of brief (< 90s.) individually-relevant stories to the PLWD (5female; age=78.2(5.3); MMSE12-29). Caregivers utilized an on-line daily log to report falls, injury falls, near falls (NFs), FPBs, and other events/issues encountered. A case series approach explored patterns of caregiver-reported FPBs and PLWD falls.

**Results:**

Caregivers logged 371 weeks (13-52) of daily reporting, 435 FPBs (12-106) and 14 PLWD falls. The first four weeks, 154 FPBs were reported (average=14(8)). Months 2 + 3 (9 weeks) yielded 148 FPBs (average=13(13). Months 3-6, 6-9, 9-12 (13 weeks each) yielded 81 FPBs (average=12(8), 29 FPBs (average=6(7) and 23 FPBs (average=5(5) respectively. Most falls or NFs, stimulated 1-4 FPBs, reducing or eliminating subsequent NFs. Six falls were caused indirectly (e.g. extraneous party created obstacle-3, caregiver extended absence-2, unrelated hospitalization-1). Eight falls related to health issues or dementia progression occurred 3+ months later.

**Conclusions:**

Family caregivers generated falls prevention ideas and encouraged PLWD falls prevention measures. A booster session may have prevented falls that occurred months after FallScape-D by inspiring FPBs matching changed circumstances. We conclude that stimulating FPBs is a viable strategy to prevent falls in PLWD.

